# A Motor-Cycle in a Country Practice

**Published:** 1912-06-08

**Authors:** 


### A MOTOR-CYCLE IN A COUNTRY PRACTICE.

### Its Specifications and Value to the General Practitioner.

The value of speed in covering the ground is being more and more appreciated every day in medical practice in country districts. How often does one feel sorry for the tired horse that, after a round of fifteen to twenty miles, has to turn out again to answer a call from five miles away on a wet or snowy day! By the help of a modern motor-cycle the distance mentioned can be covered in from ten to fifteen minutes as against forty-five to sixty minutes with horse and trap, and one need not get any wetter than one would in the ordinary dog-cart.

The writer has had a great deal of experience in the use of a motor-cycle for medical work, and he thinks it might be advisable to put on record a kind of specification of the requirements that must be fulfilled if the motor-cycle is going to be of real use to its possessor, and not turn him into such a "sight" as one can see any day touring on the main roads in summer.

### A Doctor's Essentials.

The motor-cycle as generally sold to the budding enthusiast is neither reliable in all weathers, nor does it protect its rider in any way from mud or rain or snow; but rather takes as great a delight in smothering him with as much mud as possible.

A good make of motor-cycle, nowadays, if conforming to the subjoined particulars, is as reliable as a horse and trap, is far less costly, and far more speedy, thus saving time on the ordinary round, and giving greater opportunities for nursing a practice, besides rendering extra calls and night work far less disagreeable.

The daily expenses of a horse and trap used constantly in all weathers is about sixpence a mile, whereas a motor-cycle is being very generously treated if allowed twopence a mile, and generally requires less than a penny a mile. A good motor-cycle conforming to this specification of extra and modified requirements should only cost about £70 to £80.

The engine should be one of proved reliability, should be about 5 h.p., and preferably a twin, as then if any fault arises in one cylinder, there is always the remaining one to get the machine home with. Transmission should be by enclosed chains right to the wheel, as there is great liability of any make of belt slipping in wintry weather, despite the assurances of the makers to the contrary.

There should be a reliable and efficient two-speed gear fitted, having the clutch mechanism actuated by the foot. There should also be a device to enable the engine whilst working at a diminished degree of compression to be started by the foot and whilst the rider is seated on the saddle.

The mud-guards should be of double the standard width, and the front one should be sufficiently long to hide the front of the tyre from view to anyone looking directly over the head of the machine. The mud is then flung back again to the road, and is not thrown up into the air for the rider to run into.

The front tyre should be at least a 3-inch one. The back tyre should be of the twin-tyre variety (necessitating a special rim) and should have twin-tyres of at least 3-inch size fitted.

The twin-tyre makes skidding in greasy or frosty weather much less likely, and also enables one to bring the motor-cycle home even if one of the rear tyres is badly punctured or burst, thus saving many a roadside repair.

Lubrication should be forced and automatic.

### Shields.

Special spring forks should be fitted to enable one to travel speedily and safely over "devilishly" rough country roads and lanes leading only to farm houses.

There should be a special shield applied to each side of the motor-cycle (like the one fitted now to the "Motosacoche") to protect the rider at every point from grease. A further shield should be fitted at right angles to this one in front of the line of the legs when on the footboard, and should extend from below the feet to the level of the middle of the thighs, being shaped to give the same clearance at its highest point as at its lowest but no more, and also being curved backwards at its outer edge a reasonable distance to afford ample protection and warmth to the legs. The magneto should occupy a protected position, and should be further safeguarded by a waterproof hood.

The finished motor-cycle should be put to the test of being driven for three hours without stopping, except for quick petrol refills, and going "all out," before being accepted or paid for; as makers are not so keen on making these special machines, and do not seem to take as much pride in them, or their results, as they do in their more ordinary standard types.

If the motor-cycle conforms to these specifications as well as being satisfactory in every way during its severe track test, the country general practitioner need have no hesitation in mounting it in his best clothes in fine weather, or covered with a mackintosh in wet, for he will be completely protected from mud and rain, and he will be quite as warm as in the good old-fashioned dog-cart.

